# Supplementary material for: Observational Study on the Prevalence of Urinary Incontinence in Female Athletes
Source: Int J Environ Res Public Health. 2021 May 24;18(11):5591. doi: 10.3390/ijerph18115591 (PMC8197179; doi:10.3390/ijerph18115591)
Supplement: Supplementary file 1 [file ijerph-18-05591-s001.zip › ijerph-1210887-SI.pdf]

ANEXO 1: Encuesta

1. Sexo
    A. Femenino.
    B. Masculino.
2. Edad:
3. ¿Pertenece a Castilla y León?
    A. Sí.
    B. No.
4. Dentro del atletismo, ¿A qué modalidad se dedica?:
5. ¿Qué edad tenía cuándo comenzó con este deporte?:
6. Cuantas horas a la semana le dedica a este deporte (entrenamiento + competiciones)
    A. Menos de 3 horas/semana.
    B. Entre 3 y 5 horas/semana.
    C. Ente 6 y 8 horas/semana.
    D. Más de 8 horas/semana.
7. ¿Presenta pérdidas de orina?
    A. Sí.
    B. No.
8. ¿Con qué frecuencia se presentan estas pérdidas?
    A. Continuamente durante el día.
    B. Varias veces al día.
    C. Más de 5 veces por semana
    D. Entre 5 y 2 veces por semana.
    E. Menos de 2 veces por semana.
    F. Nunca.
9. ¿Cuánta cantidad de orina cree que se le escapa?
    A. Nada.
    B. Muy poca, alguna gotita.
    C. Cantidad moderada.
    D. Mucha cantidad.
10. ¿Cuándo pierde orina?
    A. Nunca.
    B. Al toser o estornudar.

C. Al realizar deporte u otros esfuerzos físicos.
D. Antes de llegar al baño.
E. Al acabar de orinar, una vez se haya vestido.
F. De forma continuada.
G. Al dormir.
H. Sin motivo evidente.

11. ¿Ha sido intervenida quirúrgicamente en la zona del suelo pélvico?
    A. No.
    B. Sí.
12. ¿Ha dado a luz alguna vez? (En caso de cesárea, señálelo en la pregunta anterior).
    A. NO.
    B. Sí.
13. ¿Ha entrado usted en la época de la menopausia?
    A. Sí.
    B. No.
14. ¿Sus problemas urinarios afectan a su vida diaria? Puede señalar varias opciones.
    A. No.
    B. Sí, en mi vida social.
    C. Sí, en el ámbito deportivo.
    D. Sí, en el ámbito doméstico.
    E. Sí, en el ámbito sexual.
15. ¿Sus problemas urinarios le crean ansiedad, depresión, preocupación?
    A. No.
    B. Sí.
16. ¿Usa algún medio de contención tipo salva-slip?
    A. Siempre, a diario.
    B. Ocasionalmente.
    C. No, pero mojo la ropa interior.
    D. No, no tengo escapes de orina.
17. ¿Presenta algún otro problema urinario o que pueda estar relacionado con la incontinencia urinaria?
    A. Sí.
    B. No.

ANEXO 2: Cuestionarios validados

King's Health Questionnaire (KHQ)

1. ¿Cómo describiría su estado de salud general en la actualidad?

   Muy bueno ☐

   Bueno ☐

   Regular ☐

   Malo ☐

   Muy malo ☐

2. ¿Hasta qué punto piensa que sus problemas urinarios afectan a su vida?

   No, en absoluto ☐

   Un poco ☐

   Moderadamente ☐

   Mucho ☐

A continuación aparecen algunas actividades diarias que pueden verse afectadas por problemas urinarios. ¿Hasta qué punto le afectan sus problemas urinarios?

Nos gustaría que contestara a todas las preguntas, pensando sólo en las 2 últimas semanas. Simplemente marque con una cruz el casillero que corresponda a su caso.

A. Limitaciones en sus actividades diarias:

3. ¿Hasta qué punto afectan sus problemas urinarios a las tareas domésticas (ej. limpiar, hacer la compra, pequeñas reparaciones, etc.)?

   No, en absoluto ☐

   Un poco ☐

   Moderadamente ☐

   Mucho ☐

4. ¿Sus problemas urinarios afectan a su trabajo o a sus actividades diarias normales fuera de casa?

   No, en absoluto ☐

   Un poco ☐

   Moderadamente ☐

   Mucho ☐

B. Limitaciones físicas y sociales:

5. ¿Sus problemas urinarios afectan a sus actividades físicas (ej. ir de paseo, correr, hacer deporte, gimnasia, etc.)?

No, en absoluto ☐

Un poco ☐

Moderadamente ☐

Mucho ☐

6. ¿Sus problemas urinarios afectan a su capacidad para desplazarse en autobús, coche, tren, avión, etc.?

No, en absoluto ☐

Un poco ☐

Moderadamente ☐

Mucho ☐

7. ¿Sus problemas urinarios limitan su vida social?

No, en absoluto ☐

Un poco ☐

Moderadamente ☐

Mucho ☐

8. ¿Sus problemas urinarios limitan su capacidad de ver o visitar a los amigos?

No, en absoluto ☐

Un poco ☐

Moderadamente ☐

Mucho ☐

C. Relaciones personales:

9. ¿Sus problemas urinarios afectan a su relación con su pareja?

No, en absoluto ☐

Un poco ☐

Moderadamente ☐

Mucho ☐

10. ¿Sus problemas urinarios afectan a su vida sexual?

No, en absoluto ☐

Un poco ☐

Moderadamente ☐

Mucho ☐

11. ¿Sus problemas urinarios afectan a su vida familiar?

No, en absoluto ☐

Un poco ☐

Moderadamente ☐

Mucho ☐

D. Emociones:

12. ¿Sus problemas urinarios le hacen sentirse deprimido/a?

No, en absoluto ☐

Un poco ☐

Moderadamente ☐

Mucho ☐

13. ¿Sus problemas urinarios le hacen sentirse preocupado/a o nervioso/a?

No, en absoluto ☐

Un poco ☐

Moderadamente ☐

Mucho ☐

14. ¿Sus problemas urinarios le hacen sentirse mal consigo mismo/a?

No, en absoluto ☐

Un poco ☐

Moderadamente ☐

Mucho ☐

E. Sueño/energía:

15. ¿Sus problemas urinarios afectan a su sueño?

No, en absoluto ☐

Un poco ☐

Moderadamente ☐

Mucho ☐

16. ¿Sus problemas urinarios le hacen sentirse agotado/a o cansado/a?

No, en absoluto ☐

Un poco ☐

Moderadamente ☐

Mucho ☐

F. ¿Con qué frecuencia se encuentra en las siguientes situaciones?:

17. ¿Lleva compresas/pañales para mantenerse seco/a?

Nunca ☐

A veces ☐

A menudo ☐

Siempre ☐

18. ¿Tiene usted cuidado con la cantidad de líquido que bebe?

Nunca ☐

A veces ☐

A menudo ☐

Siempre ☐

19. ¿Se cambia la ropa interior porque está mojado/a?

Nunca ☐

A veces ☐

A menudo ☐

Siempre ☐

20. ¿Está preocupado/a por si huele?

Nunca ☐

A veces ☐

A menudo ☐

Siempre ☐

21. ¿Se siente incómodo/a con los demás por sus problemas urinarios?

Nunca ☐

A veces ☐

A menudo ☐

Siempre ☐

Nos gustaría saber cuáles son sus problemas urinarios y hasta qué punto le afectan. De lista siguiente elija sólo aquellos problemas que usted tenga en la actualidad y márquel con una cruz, deje sin contestar los que no correspondan a su caso.

G. ¿Hasta qué punto le afecta?:

22. Frecuencia: ir al baño muy a menudo.

Un poco ☐

Moderadamente ☐

Mucho ☐

23. Nicturia: levantarse durante la noche para orinar.

Un poco ☐

Moderadamente ☐

Mucho ☐

24. Urgencia: un fuerte deseo de orinar difícil de controlar.

Un poco ☐

Moderadamente ☐

Mucho ☐

25. Incontinencia por urgencia: escape de orina asociado a un fuerte deseo de orinar.

Un poco ☐

Moderadamente ☐

Mucho ☐

26. Incontinencia por esfuerzo: escape de orina por actividad física (ej. toser, estornudar, correr, etc.).

Un poco ☐

Moderadamente ☐

Mucho ☐

27. Enuresis nocturna: mojar la cama durante la noche.

Un poco ☐

Moderadamente ☐

Mucho ☐

28. Incontinencia en el acto sexual: escape de orina durante el acto sexual (coito).

Un poco ☐

Moderadamente ☐

Mucho ☐

29. Infecciones frecuentes en las vías urinarias:

Un poco ☐

Moderadamente ☐

Mucho ☐

30. Dolor en la vejiga:

Un poco ☐

Moderadamente ☐

Mucho ☐

31. Dificultad al orinar:

Un poco ☐

Moderadamente ☐

Mucho ☐

32. Otro problema urinario (especifique): ....

Un poco ☐

Moderadamente ☐

Mucho ☐

Por favor, compruebe que ha contestado a todas las preguntas. ¡Muchas gracias!

International Consultation on Incontinence Questionaire-Urinary Short Form (ICQ-UI SF)

1. Usted es (señale cuál): Mujer Varón

2. ¿Con qué frecuencia pierde orina? (marque una):

1 Nunca

2 Una vez a la semana o menos

3 Dos o tres veces a la semana

4 Una vez al día

5 Varias veces al día

6 Continuamente

3. Nos gustaría saber su impresión acerca de la cantidad de orina que usted cree que se le escapa. Cantidad de orina que pierde habitualmente (tanto si lleva protección como si no). (Marque una)

1 No se me escapa nada

2 Muy poca cantidad

3 Una cantidad moderada

4 Mucha cantidad

4. Estos escapes de orina que tiene ¿cuánto afectan su vida diaria? Por favor marque una cruz, en la siguiente lista, un número entre 0 (no me afectan nada) y 10 (me afectan mucho):

0 1 2 3 4 5 6 7 8 9 10

Nada Mucho

Puntuación de ICI-Q: sume las puntuaciones de las preguntas 2+3+4:

5. ¿Cuándo pierde orina? (señale todo lo que le pasa a usted)

1 Nunca pierde orina

2 Pierde orina antes de llegar al WC

3 Pierde orina cuando tose o estornuda

4 Pierde cuando duerme

5 Pierde orina cuando hace esfuerzos físicos/ejercicio

6 Pierde orina al acabar de orinar y ya se ha vestido

7 Pierde orina sin motivo evidente

8 Pierde orina de forma continua
